# Improved Thermoelectric Properties of SrTiO_3_ via (La, Dy and N) Co-Doping: DFT Approach

**DOI:** 10.3390/molecules27227923

**Published:** 2022-11-16

**Authors:** Pornsawan Sikam, Ruhan Thirayatorn, Thanayut Kaewmaraya, Prasit Thongbai, Pairot Moontragoon, Zoran Ikonic

**Affiliations:** 1 Research Center for Quantum Technology, Faculty of Science, Chiang Mai University, Chiang Mai 50200, Thailand; 2 Office of Research Administration, Chiang Mai University, Chiang Mai 50200, Thailand; 3 Department of Physics, Khon Kaen University, Khon Kaen 40002, Thailand; 4 Institute of Nanomaterials Research and Innovation for Energy (IN-RIE), Research Network of NANOTEC-KKU (RNN), Khon Kaen University, Khon Kaen 40002, Thailand; 5 Thailand Center of Excellence in Physics, Commission on Higher Education, Bangkok 10400, Thailand; 6 School of Electronic and Electrical Engineering, University of Leeds, Leeds LS2 9JT, UK

**Keywords:** thermoelectric properties, SrTiO_3_, rare-earth doping

## Abstract

This work considers the enhancement of the thermoelectric figure of merit, ZT, of SrTiO_3_ (STO) semiconductors by (La, Dy and N) co-doping. We have focused on SrTiO_3_ because it is a semiconductor with a high Seebeck coefficient compared to that of metals. It is expected that SrTiO_3_ can provide a high power factor, because the capability of converting heat into electricity is proportional to the Seebeck coefficient squared. This research aims to improve the thermoelectric performance of SrTiO_3_ by replacing host atoms by La, Dy and N atoms based on a theoretical approach performed with the Vienna Ab Initio Simulation Package (VASP) code. Here, undoped SrTiO_3_, Sr_0.875_La_0.125_TiO_3_, Sr_0.875_Dy_0.125_TiO_3_, SrTiO_2.958_N_0.042_, Sr_0.750_La_0.125_Dy_0.125_TiO_3_ and Sr_0.875_La_0.125_TiO_2.958_N_0.042_ are studied to investigate the influence of La, Dy and N doping on the thermoelectric properties of the SrTiO_3_ semiconductor. The undoped and La-, Dy- and N-doped STO structures are optimized. Next, the density of states (DOS), band structures, Seebeck coefficient, electrical conductivity per relaxation time, thermal conductivity per relaxation time and figure of merit (ZT) of all the doped systems are studied. From first-principles calculations, STO exhibits a high Seebeck coefficient and high figure of merit. However, metal and nonmetal doping, i.e., (La, N) co-doping, can generate a figure of merit higher than that of undoped STO. Interestingly, La, Dy and N doping can significantly shift the Fermi level and change the DOS of SrTiO_3_ around the Fermi level, leading to very different thermoelectric properties than those of undoped SrTiO_3_. All doped systems considered here show greater electrical conductivity per relaxation time than undoped STO. In particular, (La, N) co-doped STO exhibits the highest ZT of 0.79 at 300 K, and still a high value of 0.77 at 1000 K, as well as high electrical conductivity per relaxation time. This renders it a viable candidate for high-temperature applications.

## 1. Introduction

SrTiO_3_ (STO), a semiconductor with a 3.2 eV [1] indirect bandgap (*E_g_*) and a perovskite structure, has received much research attention. This is due to its outstanding properties. Researchers are presently trying to improve its properties, such as bandgap width, electrical conductivity, thermal conductivity, and effective mass, to enhance the performance of STO in applications such as dye-sensitized solar cells [2,3,4], photocatalysis [5,6,7], water splitting [8,9], hydrogen production [10] and thermoelectric devices [11,12,13]. There is growing interest in using STO as a catalyst for photocatalytic processes because of its semiconducting properties, thermal stability and photocorrosion resistance. However, the bandgap of this oxide corresponds to UV light, which is only a minor portion (around 4%) of the entire solar spectrum. This factor significantly limits the photocatalytic performance of STO. A possible way to enhance the efficiency of this system is engineering the bandgap by introducing impurities so that it appropriately matches the energy of visible and infrared light.

Considering the thermoelectric properties of STO, Sun and Singh [14] showed in numerical results in 2016 that the ZT of undoped STO is 0.7 at 1400 K. This value was calculated using BoltzTraP code. A number of researchers attempted to engineer the electronic properties and to enhance the thermoelectric properties of STO in various ways. These approaches included both monoatomic and multiatomic co-doping, creating defects by removing some host atoms, and designing heterostructures [15,16,17,18,19,20]. Various elements were chosen for the A and B sites of ABO_3_, symmetrically replacing Sr and Ti sites with elements such as La [11,21,22], Zn [23], Pr [15], Nd [15], Sm [15], Li [24], K [24], Be [24], Dy [25], Y [26], and Nb [27,28]. Doping with these elements can improve the electronic and thermoelectric performance of STO, especially (La, Dy, Nb) co-doping [20]. Doping can improve electrical conductivity and decrease thermal conductivity. The largest experimentally measured ZT values are 0.28, when x = 0.05, and 0.27, when x = 0.2, at 1100 K. However, some unconventional elemental doping can successfully improve the electronic and electrical properties of STO. For instance, in 2022, Fadlallah and Gogova [16] reported theoretical calculations of structural, electronic, magnetic, optical and photocatalytic property changes with (La, X) and (Y, M) co-doping, where X can be Al, Sc, Cr, Mn, Fe, Co, Ni, or Mo, and M can be Al, Cr, or Mo on STO. Most X and M mono-doping gives a smaller bandgap than co-doped systems, such as (La, Ni) co-doping. Moreover, La and Y doping, as well as (La, Y, Al) and (La, Sc) co-doping do not enhance the conductivity of STO, while other mono-dopants and co-dopants can improve it. There has been a successful improvement in the thermoelectric properties of STO when doped with elements with various oxidation numbers (La, Dy and N) [21,25,29,30]. Our previous theoretical work has shown that N doping can greatly improve the Seebeck coefficient and figure of merit. The current computational study aimed to elucidate the thermoelectric properties of (La, Dy, N) co-doped STO.

Inspired by the problem of the limited ZT of STO, here we go beyond mono-doping to improve its thermoelectric performance by La, Dy, and N co-doping of the STO semiconductors. We undertake a computational study of (La, Dy, N) doping of SrTiO_3_, and find that the thermoelectric properties, especially the ZT, of STO with (La, N) co-doped material, can be significantly improved. Appropriate atom doping can overcome high electrical conductivity and thermal conductivity during relaxation. Co-dopants provide very large Seebeck coefficients, higher than that of undoped and mono-doped STO. The co-doped systems show very high figures of merit. These results come from a larger number of carriers in the STO when various elements with different oxidation numbers are doped into it. An increased number of carriers improves the electronic properties, which can be clearly seen in the DOS and band structures, e.g., through the creation of a new stepping stone in the bandgap and Fermi level positions. This leads, in turn, to greatly improved thermoelectric properties.

## 2. Methodology

In this theoretical study, the structures of undoped STO and (La, Dy and N)-doped SrTiO_3_ were modeled as 2 × 2 × 2 super-cells, eight times as large as the size of a primitive unit cell. The N (2s^2^2p^3^), Sr (4s^2^4p^6^5s^2^), Ti (3p^6^4s^2^3d^2^) and O (2s^2^2p^4^) orbitals were treated as valence electrons. Kr4d and Xe4 were treated as core electrons for La and Dy, respectively. The doping amounts of La, Dy and N are shown in Table S1. Calculations were performed using the Vienna Ab Initio Simulation Package (VASP) with 27 × 27 × 27 k-point meshes in the Brillouin zone and a 500-eV cutoff energy for plane waves. Projector augmented waves (PAW) [31] within the Perdew–Burke–Ernzerhof (PBE) generalized gradient approximation with Hubbard parameters (GGA+U) were employed with a Coulomb interaction *U* of 8.7 eV for Ti, and 6.0 eV for both La and Dy. After that, semiclassical Boltzmann transport theory utilizing the BoltzTraP code [32] was used to calculate the thermoelectric properties. Finally, the VASPKIT code was utilized to extract the raw data from the VASP calculations [33].

## 3. Results and Discussion

**Figure 1:**
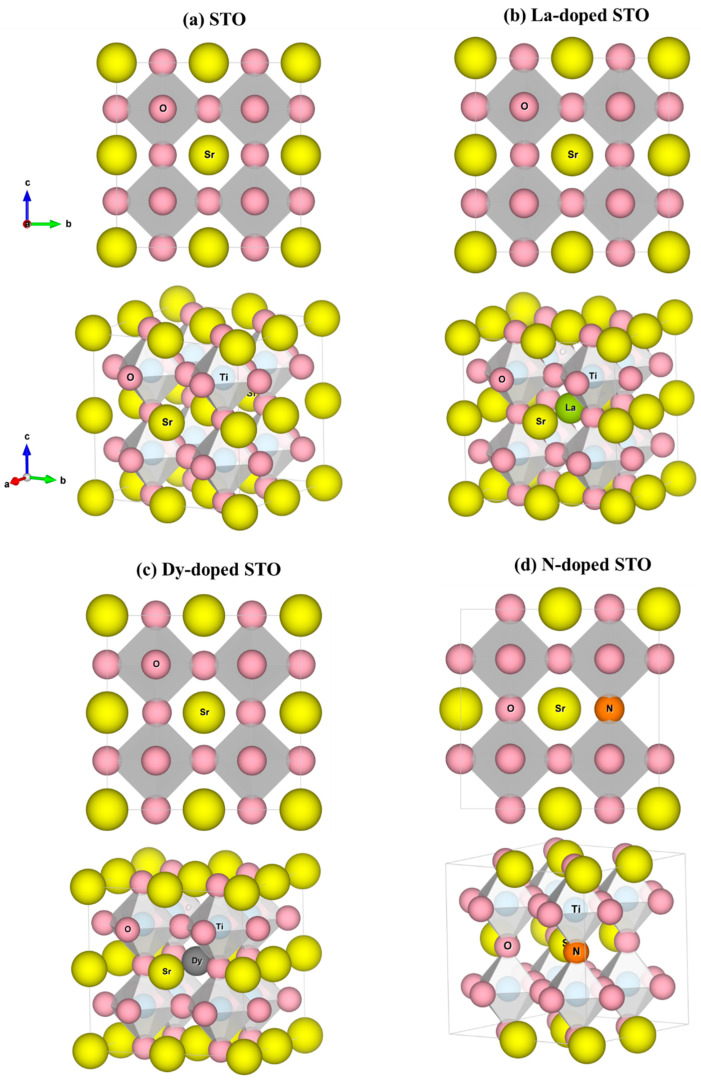
Optimized structures of (**a**) undoped SrTiO_3_, (**b**) La-doped SrTiO_3_, (**c**) Dy-doped SrTiO_3_ and (**d**) N-doped SrTiO_3_. Yellow spheres represent Sr atoms, blue are Ti, pink are O, green are La, grey are Dy, and orange are N.

**Figure 2:**
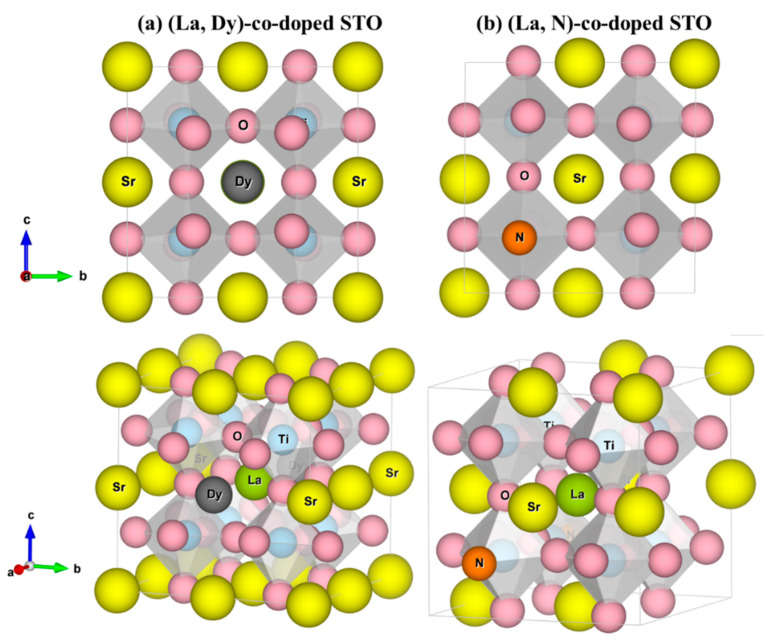
Optimized structures of (**a**) (La, Dy) co-doped SrTiO_3_ and, (**b**) (La, N) co-doped SrTiO_3_. Yellow, blue, pink, green, gray and orange spheres represent Sr, Ti, O, La, Dy and N, respectively.

The optimized structure of pure SrTiO_3_ is shown in Figure 1a. The cell contains forty atoms; eight Sr, eight Ti and twenty-four O atoms. The ionic radii of Sr^+2^, Ti^+4^, and O^−2^ are 1.26, 0.61 and 1.40 Å, respectively, and those of N^−3^, Dy^+3^, and La^+3^ are 1.71, 1.03 and 1.16 Å, respectively. The total energy of the host-site replacement with mono-doped atoms is calculated to predict which host atoms can be replaced by La, Dy and N atoms. The results are given in Table S2 and Figures S1–S5. It is acknowledged that the choices of atomic sites of the dopants play a deciding role in the properties. For instance, there are 8 × 24 = 192 possibilities in (La, N)-co-doped SrTiO_3_ because of 8 Sr and 24 O atoms in the modeled cell that have been replaced by 1 La and 1 N atoms, respectively. Nevertheless, the structures considered in our theoretical study are a few exemplified cases out of the entire range of possibilities that are capable of offering improved thermoelectric functionality. In a primitive unit cell of SrTO_3_ containing five atoms—one atom of Sr, one atom of Ti and three atoms of O—all the Sr and Ti atoms for the 2 × 2 × 2 supercell are in the same environment because of the symmetric crystallography. Thus, replacing an atom of La or Dy in a central-Sr site in the supercell, as shown in Figure 2, leads to the same Sr-host atoms (seven atoms) because of the symmetry.

The lowest formation energy (*E_form_*), predicting the most stable structures in the experiments, is observed when Sr atoms are replaced by La and Dy and O atoms are replaced by N. Since the ionic radius of Sr^2+^ is larger than that of both Dy^3+^ and La^3+^, it causes the La and Dy replacement on the Sr sites to provide a more stable structure compared to the Ti site. For the O site, La and Dy are undesirable as replacement for the O site because of the difference in physical properties, nonmetal gas (O^2−^) and metal solid (La^3+^ and Dy^3+^), although the ionic radius of O^2−^ is also larger than the La^3+^ and Dy^3+^. Corresponding to the lowest *E_form_* for N replacing an O site, both are non-metals. Thus, in this calculation, the Sr site in SrTiO_3_ will be replaced by La and Dy and referred to as La-doped SrTiO_3_ (La-STO) and Dy-doped SrTiO_3_ (Dy-STO), respectively. An atom of O will be replaced by N, referred to as N-doped SrTiO_3_ (N-STO). Also, (La, Dy)-doped SrTiO_3_ and (La, N)-doped SrTiO_3_ are labeled as (La, Dy)-STO and (La, N)-STO, respectively. Thus, all the systems studied in this computational work are SrTiO_3_, Sr_0.875_La_0.125_TiO_3_, Sr_0.875_Dy_0.125_TiO_3_, SrTiO_2.958_N_0.042_, Sr_0.875_La_0.125_Ti_0.875_Dy_0.125_O_3_ and Sr_0.875_La_0.125_Ti O_2.958_N_0.042_. All optimized structures of (La, Dy, N) mono-doped SrTiO_3_ are shown in Figure 1b–d, while optimized structures of the co-doped systems are shown in Figure 2. Moreover, lattice parameters of the optimized structures are depicted in Table S3.

**Figure 3:**
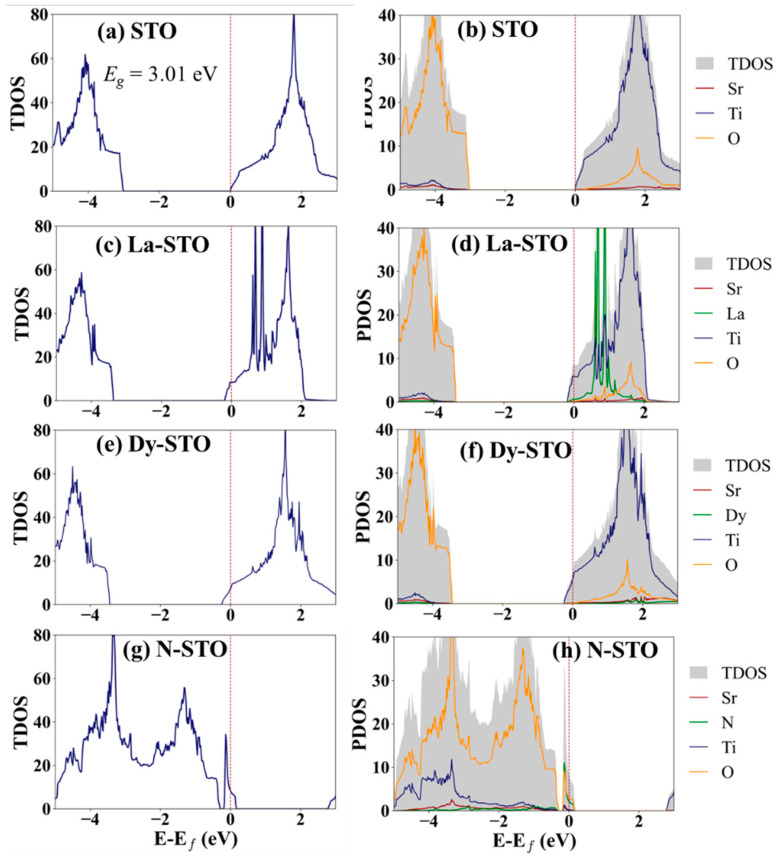
(**a**,**c**,**e**,**g**) Total and (**b**,**d**,**f**,**h**) partial DOS of (**a**,**b**) undoped SrTiO_3_ and for mono-doping with: (**c**,**d**) La, (**e**,**f**) Dy, and (**g**,**h**) N.

The total density of states (TDOS) of the undoped SrTiO_3_ is illustrated in Figure 3a. Red dashed lines at x = 0 represent Fermi levels. The Fermi level (*E_f_*) is 7.27 eV, and this is the conduction band minimum. Therefore, undoped SrTiO_3_ exhibits n-type behavior, in agreement with experimental measurements and other literature reports [34,35].

After La doping, the highest state that the carriers occupy is the ground state or Fermi level (*E_f_*), located at the bottom of the conduction band, at 8.00 eV, as seen in Figure 3c,d. *E_f_* shifts to higher energy compared to undoped SrTiO_3_. This is caused by an increase of the number of electrons in the system when La is doped into SrTiO_3_. The electron configuration of Sr is Kr 5s^2^, while that of La is Kr 4d^10^ 5s^2^ 5p^6^ 5d^1^ 6s^2^. In other words, the oxidation number of La is +3, while it is +2 for Sr. Thus, La doping is electron doping. Likewise, *E_f_* shifts to 7.79 eV with electron doping for Dy doping on the Sr site because Dy and La have the same oxidation number as previously mentioned. For N doping, the Fermi level shifts from the conduction band to the valence band and new states are found around the valence band maximum in the N mono-doped structures. The Fermi level of N-doped SrTiO_3_ is 4.48 eV. The Fermi energy shifts to a lower level because oxygen substituted by nitrogen is a p-type doping or hole doping and the number of electrons is reduced. The electron configurations of O and N are He 2s^2^ 2p^4^ and He 2s^2^ 2p^3^, respectively. Notably, from the PDOS graphs, O and Ti atoms contribute the valence band maximum and conduction band minimum for all mono-doping systems.

**Figure 4:**
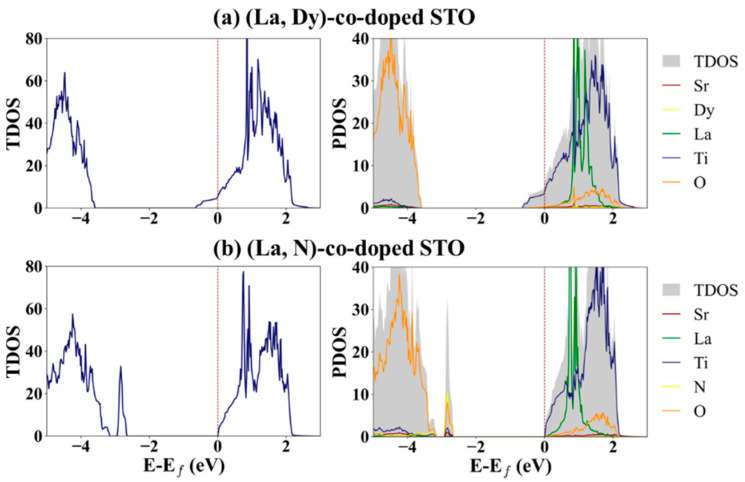
TDOS of (**left**) (La, Dy) co-doped SrTiO_3_ and, (**right**) (La, N) co-doped SrTiO_3_ with dashed lines denoting *E_f_*, located at 6.27 and 5.39 eV for (La, Dy) co-doped STO and (La, N) co-doped STO, respectively.

For co-doped systems, the total DOS of (La, Dy) and (La, N) co-doped SrTiO_3_ structures is shown in Figure 4. E_f_ is shifted to a higher level compared to the STO. The *E_f_* is at 8.22 and 7.78 eV for (La, Dy) and (La, N) co-doping, respectively. Comparing the DOS of the co-doped systems to those of undoped SrTiO_3_, it can be seen that (La, N) co-doping can induce a new state in the forbidden band, like the N doping. For the DOS of (La, Dy)-co-doping, not much difference is observed compared to the La and Dy mono-doping. A clear difference in electronic structures is seen for both mono-doping and co-doping, which affects the carrier behavior and is expected to be able to improve the thermoelectric properties of the STO.

Partial and projected DOS (PDOS) plots are shown in Figures S6–S15. The valence band maximum comes from the *p* electrons of Sr and O while the conduction band minimum results from the *d* orbitals of Ti for STO. After doping, a clear change in the DOS of valence orbitals for the host atoms is observed for N mono- and (La, N) co-doping. New states at the valence band maximum (VBM), called “stepping stones,” are observed for the systems with N doping. These stepping stones form from interaction of all elements in the systems. Moreover, interestingly, Ti states are at the bottom of the CBM for STO and (La, N)-co-doped STO, the same position as *E_f_*, while they are lower than the Fermi level for La-, Dy- and (La, Dy)-doped STO. (La, N)-doped STO is a neutrally doped material, similar to the undoped STO, because doped atoms create one hole and one free electron. It therefore possesses similar Ti states, at the CMB, to STO.

**Figure 5:**
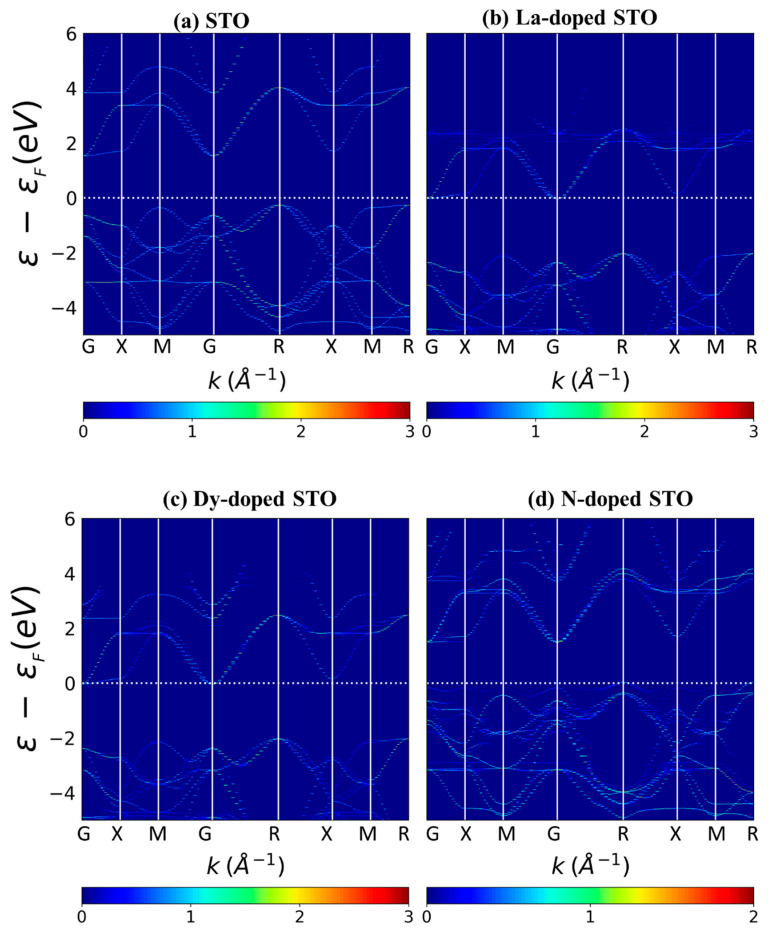
Band structures of (**a**) undoped SrTiO_3_, (**b**) La-doped SrTiO_3_, (**c**) Dy-doped SrTiO_3_ and (**d**) N-doped SrTiO_3_, where white dashed lines denote the Fermi level, set to zero. The DOS scale is shown at the bottom of each figure.

**Figure 6:**
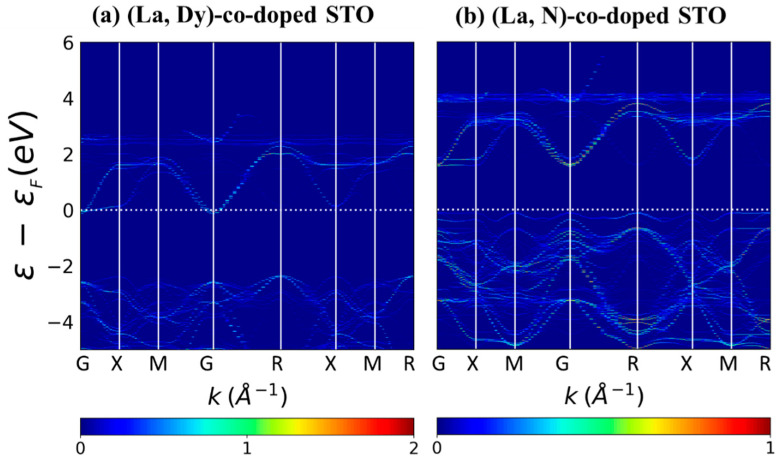
Band structure of (La, N)-co-doped SrTiO_3_. White dashed lines represent the Fermi level, set to zero, and the DOS scale is shown at the bottom of the figure.

The unfolding band structures of undoped and doped SrTiO_3_ were calculated, and the results are shown in Figure 5 and Figure 6. For the undoped system, the VBM is at the R point, while the CBM is at the Г point. So, SrTiO_3_ is found to be an indirect bandgap material, in agreement with previous experimental and theoretical reports [36,37]. Similar to SrTiO_3_, La-doped SrTiO_3_ and Dy-doped SrTiO_3_ show an indirect bandgap. The results we obtain from the band structure calculations are similar to those obtained from the DOS. N-doped SrTiO_3_ has states above the valence band near E_f_. New states above the valence band are found for N doping, leading to a narrower bandgap than undoped SrTiO_3_, in agreement with the previous theoretical and experimental studies [38,39]. This step above the valence band is also observed in (La, N)-co-doped systems, corresponding to the DOS.

From the study of electronic properties, we can conclude that the dopant atoms added into SrTiO_3_ can change the electronic behavior, as predicted from the DOS and band structure of SrTiO_3_. From the DOS we can group the systems for this study as STO and (La, N)-co-doped STO are the n-type semiconductors while N-STO is the p-type semiconductor. La-, Dy-, and (La, Dy)-doped STO are metallic materials. Due to the significant change in DOS after La, Dy and N atoms are added into the STO, it is possible that all systems, especially N-STO and (La, N)-co-doped STO, will show different thermoelectric properties because of the change in the number of carriers in the materials.

**Figure 7:**
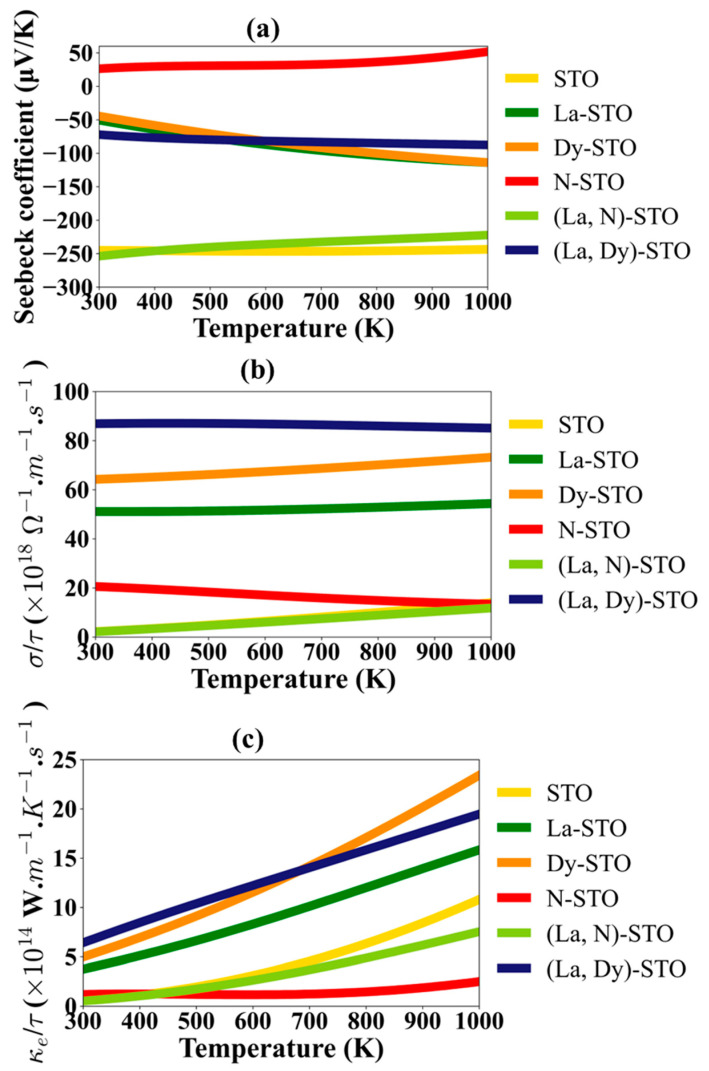
(**a**) Seebeck coefficient, (**b**) ratio of electrical conductivity to relaxation time, and, (**c**) ratio of the electronic thermal conductivity to relaxation time of undoped and doped STO.

**Figure 8:**
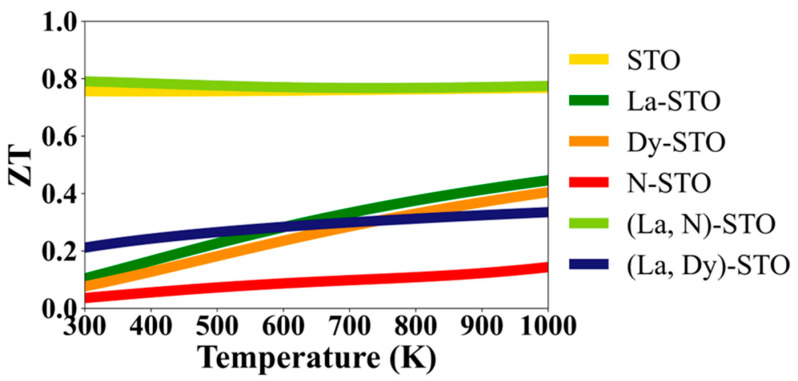
Dimensionless figure of merit of STO and doped STO.

Thermoelectric properties calculated using the BoltzTraP code are shown in Figure 7, Figure 8 and Figures S16–S19. As seen in Figure 7a, almost all materials, except N-doped STO, show a negative Seebeck coefficient, making them n-type semiconductors, in agreement with the DOS results and a previous experimental report [14]. N-doped STO is a p-type semiconductor with positive Seebeck coefficient. Undoped STO and (La, N)-co-doped STO exhibit Seebeck coefficients with large magnitudes of more than 200 µV/K and they remain almost constant along the temperature change from 300–1000 K, with about 3 and 30 µV/K changes when the temperature reaches 1000 K for STO and (La, N)-co-doped STO, respectively. At room temperature, Seebeck coefficients of undoped STO and (La, N)-co-doped STO are −245.07 and −253.86.23 µV/K, respectively, and are −243.61 and −222.23 µV/K at 1000 K. For La-, Dy-, N-, and (La, Dy)-doped STO, the magnitudes of the Seebeck coefficient are less than 100 µV/K at room temperature (300 K). These are 50.47, 44.45, 26.34 and 72.17 µV/K for La-, Dy-, N-, and (La, Dy)-doped STO, respectively. However, for the temperature increase to 1000 K, the magnitudes of the Seebeck coefficient increase for La-, Dy, and (La, Dy)-doped STO. These are 113.96, 113.78 and 87.56 µV/K, respectively. Opposite to N-doped STO, the magnitudes of the Seebeck coefficient decrease with an increase in temperature and drop to 26.34 µV/K at 1000 K. Therefore, STO and (La, N) co-doped STO are promising materials that exhibit the greatest capability to transform a temperature gradient into an electromotive force or voltage.

Figure 7b shows the ratio of an electrical conductivity to relaxation time (σ/τ) for the STO and doped STO materials. STO and (La, N)-co-doped STO have the smallest σ/τ, opposite to the Seebeck coefficient. The highest σ/τ is for (La, Dy)-co-doped STO, followed by Dy- and La-doped STO. Although strange atom doping cannot improve the magnitude of the Seebeck coefficient of STO, La, Dy, and N can efficiently improve the electrical conductivity compared to undoped STO. This means that an increase in number of carriers for both hole and electron doping can improve carrier mobility and support the electrical conductivity when the relaxation time is fixed. The reason for the greater Seebeck coefficient but lower electrical conductivity per relaxation time in STO and (La, N)-co-doped STO compared to the other doping systems is that both STO and (La, N)-co-doped STO are semiconductors. E_f_ is at the bottom of CBM, while the La-, Dy-, N- and (La, Dy)-doped STO shows metallic behavior, as seen in the DOS. Normally, metals have high electrical conductivity but low Seebeck coefficients, while semiconductors have poorer electrical conductivity but higher Seebeck coefficients compared to metals. Since the Seebeck coefficient is functional to mobility, semiconductors have better Seebeck coefficient than the metals [40].

The ratio of electronic thermal conductivity to relaxation time (κ_e_/τ) trends are the same as for σ/τ. This is because both κ_e_/τ and σ/τ variations are effects that arise from electron motion. It was observed that STO, N-STO and (La, N)-co-doped STO show a small value of κ_e_/τ while La-STO, Dy-STO and (La, Dy)-co-doped STO show larger values. Clearly, doping with N or (La, N) can reduce the electronic thermal conductivity. It therefore seems like N plays a more important role than La and Dy in changing the carrier mobility, including the electrical and thermal conductivity of STO.

The numerical results obtained in this study are in agreement with Chen et al. [17]. They reported theoretical results that La mono-doping provided a negative Seebeck coefficient, similar to undoped SrTiO_3_ as well as the small negative value (−50 μV/K) of the Seebeck coefficient being obtained from the calculations [17]. Comparing the Seebeck coefficients reported by Chen et al. [17] and those found in our work, the differences result from the methodology in the calculation. The energy cutoff of 600 eV, with GGA and k-points of 9 × 9 × 24, were employed in the calculation [17], while an energy cutoff of 500 eV, with GGA+U in PBE and k-points of 27 × 27 × 27, are treated in our work. Normally, the number of k-points is sensitive to the Fermi level, resulting in the change to transport properties of the carriers. The enhancement in electrical conductivity after La is doped into STO is also found in an experiment reported by Lv [41]. They revealed that the electrical conductivity of 20% La doping into STO is the highest value compared to 10%, 30% and 40% La doping. This results from an electric neutral imbalance of replacing Sr^2+^ by La^3+^. It therefore affects the oxidation number of Ti and conductivity [41].

The thermal to electrical energy conversion is described by the figure of merit, ZT. By definition, *ZT = S^2^σT/κ*, where *T* is the temperature, *S* is the Seebeck coefficient, *σ* is the electrical conductivity, and *κ* is the thermal conductivity. The ZT values reported here are calculated for electronic thermal conductivity only, and do not include lattice thermal conductivity. Figure 8 shows the ZT of doped and undoped STO calculated using σ/τ and κ_e_/τ. However, since the relaxation times for *σ* and *κ_e_* are equal, the factors of τ cancel out, and the ZT is independent of this value. The maximum values of ZT for STO, La-STO, Dy-STO, N-STO, (La, Dy)-STO and (La, N)-STO are 0.77, 0.45, 0.40, 0.14, 0.33, and 0.79, respectively. The ZT of (La, N)-co-doped STO is better than for undoped STO. The huge ZT of undoped STO and (La, N)-co-doped STO results from the massive magnitude of Seebeck coefficients, as previously mentioned. Here, the most interesting material is (La, N)-co-doped STO. It shows a ZT value in the range 0.77–0.79 in the temperature interval of 300–1000 K, which is always nigher than that of undoped STO at all temperatures. Interestingly, even though their ZT values are less than that of STO, the ZT values of La- and Dy-doped STO increase with temperatures up to 1000 K.

Considering the previous experimental results, Lu et al. [42] experimentally studied the thermoelectric properties of STO and La-doped STO ceramics, Sr_1−3x/2_La_x_TiO_3−δ_ for x in the range 0.10–0.30. The magnitude of the Seebeck coefficient of undoped STO is 300–400 μV/K at temperatures of 450–1000 K, close to our theoretical results. After La is doped into the STO ceramic, the measured magnitude of the Seebeck coefficient is smaller than that of the undoped STO, exhibiting the same trend as the theoretical results obtained in our work. For electrical conductivity, the smallest values are observed in the undoped STO compared to the La-doped STO for temperatures in the range 450–1000 K [42]. The electrical conductivity measured in the experiment also shows the same trend as the electrical conductivity per relaxation time obtained from the theoretical results of our work. In the case of thermal conductivity, the experiment revealed that the highest thermal conductivity is observed in undoped STO compared to La-doped STO, the opposite trend to that predicted by the theoretical calculations of our work. It is possible that only the electronic thermal conductivity is included in the calculation. Phonon or heat transfer caused by lattice vibrations is not included in our predictions, while it is included in the experiment.

The ZT of the STO observed from the experiment is less than 0.05 and this value is quite constant during an increase in temperatures [42]. The trends and values of the ZT obtained from experimental and theoretical approaches in our work are similar. Comparing the ZT of (La, Dy)-co-doping in experimental and theoretical studies, the experiment revealed that ZT values rise with an increase in the temperature from 300 to 900 K [20]. After that, it is almost constant when the temperature increases, and reaches of 0.22 at 1000 K [20]. These experimental results are quite close to the computational results found in our work. Specifically, here the ZT of (La, Dy)-co-doped STO is predicted to increase with an increase in temperature between 300–1000 K. The ZT starts from 0.21 at 300 K and it continually increases until reaching 0.33 at 1000 K. Because our theoretical results are corresponding to experiments, our theoretical approach is based on the VASP and BoltzTrap codes which can provide reliable results to guide experimentalists in improving the electronic and thermoelectric properties of semiconductors.

From the ZT values calculated in our theoretical study, (La, N)-co-doped STO is the best potential candidate for thermoelectric applications. These results reveal that co-doping with metal and nonmetal (La and N) can significantly improve the thermoelectric performance of STO. Notably, metal–nonmetal doping can improve the ZT of STO more than metal–metal (La and Dy) doping. These improved thermoelectric properties result from an increased number of carriers for both electrons and holes in the STO. A larger number of carriers means greater electron motion, resulting in higher thermal to electrical energy conversion. The reason that (La, N)-co-doped STO possesses a large value of the ZT is that it has a large Seebeck coefficient. Additionally, the high absolute value of the Seebeck coefficient results from a large difference in the number of carriers in the system. La and N co-doping of STO can improve the number of carriers because electrons and holes are the majority of carriers for La and N when they respectively replace Sr and O. Thus, when La and N are doped into STO, added carriers greatly affect the host material. However, lattice thermal conductivity should be included in further studies because it will make the results more reliable.

## 4. Conclusions

In this study, we aimed to improve the thermoelectric properties of STO via co-doping of La, Dy and N. We studied undoped and (La, Dy and N)-doped STO, using the first-principles approach to calculate the DOS, band structures and thermoelectric properties. To model the STO and doped STO supercells, the formation energy and the total energy for replacing the strange atoms by different host atoms were studied to examine the most stable structures and the possibility to synthesize these materials in the experiments. From the total DOS and band structure results, we found that appropriate atom doping can shift the Fermi level and change the electronic band structures, resulting in enhancement of the thermoelectric properties of STO due to a greater number of charge carriers in the system. Very large Seebeck coefficients and a very low ratio of thermal conductivity to relaxation time were investigated for (La, N)-co-doping into STO. The thermoelectric performance of the co-doped systems is better than that of the undoped STO. Thus, it can be concluded that our work successfully predicts the improved thermoelectric performance and figure of merit, ZT, as a result of (La and N) doping of STO. The best material is predicted to be (La, N)-co-doped STO. It has a ZT value of 0.79 which is greater than that of undoped STO and still exhibits a high ZT at 1000 K. The work demonstrates the successful synergy of La and N in improving the thermoelectric properties of the STO, which has not been reported before. This can help guide experimental and theoretical research to reach ultrahigh ZT and to produce efficient thermoelectric devices for applications in the modern world.

## Data Availability

Not applicable.

## Supplementary Materials

The following supporting information can be downloaded at: https://www.mdpi.com/article/10.3390/molecules27227923/s1, Figure S1: The optimized structures of La-doped SrTiO_3_ after replacing La atoms in Sr, Ti, and O sites with formation energy (E_form_) as the total energy as listed in Table S2. The pink spheres are the O atoms, yellow are Sr, light blue are Ti, and green are La. E_min_ indicates the structure providing the lowest formation energy. Figure S2: The optimized structures of Dy-doped SrTiO3 after replacing Dy atoms in Sr, Ti, and O sites with formation energy (E_form_) as the total energy as listed in Table S2. The pink spheres are the O atoms, yellow are Sr, light blue are Ti, and grey are Dy. E_min_ indicates the structure providing the lowest formation energy. Figure S3: The optimized structures of N-doped SrTiO3 after replacing N atoms in Sr, Ti, and O sites with formation energy (E_form_) as the total energy as listed in Table S2. The pink spheres are the O atoms, yellow are Sr, light blue are Ti, and orange are N. E_min_ indicates the structure providing the lowest formation energy. Figure S4: Three different La-N distances, with the total energy of the supercell configuration, for 3 different sites of La and N doping, in which both La and N atoms replace Sr and O sites, respectively. E_min_ indicates the structure providing the lowest total energy, which is the most stable structure. Figure S5: Three different La-Dy distances, with the total energy of the supercell configuration, for 3 different sites of La and Dy doping, in which both La and Dy atoms replace Sr sites. E_min_ indicates the structure providing the lowest total energy, which is the most stable structure. Figure S6: DOS of *s* orbitals of Sr for (a) undoped, (b) La-doped, (c) Dy-doped, (d) N-doped, (e) (La, Dy)-doped, and (f) (La, N)-doped SrTiO_3_. Figure S7: DOS of p orbitals of Sr for (a) undoped, (b) La-doped, (c) Dy-doped, (d) N-doped, (e) (La, Dy)-doped, and (f) (La, N)-doped SrTiO_3_. Figure S8: DOS of s orbitals of Ti for (a) undoped, (b) La-doped, (c) Dy-doped, (d) N-doped, (e) (La, Dy)-doped, and (f) (La, N)-doped SrTiO_3_. Figure S9: DOS of p orbitals of Ti for (a) undoped, (b) La-doped, (c) Dy-doped, (d) N-doped, (e) (La, Dy)-doped, and (f) (La, N)-doped SrTiO_3_. Figure S10: DOS of *d* orbitals of Ti for (a) undoped, (b) La-doped, (c) Dy-doped, (d) N-doped, (e) (La, Dy)-doped, and (f) (La, N)-doped SrTiO_3_. Figure S11: DOS of *s* orbitals of O for (a) undoped, (b, c) La-doped, (d, e) Dy-doped, (f, g) N-doped, (h, i) (La, Dy)-doped, and (j, k) (La, N)-doped SrTiO_3_. Two O atoms are observed at positions which are (a, b, d, f, h, and j) remote from and (c, e, g, i, and k) neighboring the doped atoms. Figure S12: DOS of *p* orbitals of O for (a) undoped, (b, c) La-doped, (d, e) Dy-doped, (f, g) N-doped, (h, i) (La, Dy)-doped, and (j, k) (La, N)-doped SrTiO_3_. Two O atoms are observed at positions which are (a, b, d, f, h, and j) remote from and (c, e, g, i, and k) neighboring the doped atoms. Figure S13: DOS of *s*, *p*, *d* and f orbitals of La for (a) La-doped, (b) (La, Dy)-doped, and (c) (La, N)-doped SrTiO_3_. Figure S14: DOS of *s*, *p*, and *d* orbitals of Dy for (a) Dy-doped and (b) (La, Dy)-doped SrTiO_3_. Figure S15: DOS of *s* and *p* orbitals of N for (a) N-doped and (c) (La, N)-doped SrTiO_3_. Figure S16: Seebeck coefficient versus temperature variation for (a) undoped, (b) La-doped, (c) Dy-doped, (d) N-doped, (e) (La, Dy)-doped, and (f) (La, N)-doped SrTiO_3_. Figure S17: Ratio of electrical conductivity to relaxation time (σ/τ) with temperature variation for (a) undoped, (b) La-doped, (c) Dy-doped, (d) N-doped, (e) (La, Dy)-doped, and (f) (La, N)-doped SrTiO_3_. Figure S18: Ratio of electronic thermal conductivity to relaxation time (κe/τ) with temperature variation for (a) undoped, (b) La-doped, (c) Dy-doped, (d) N-doped, (e) (La, Dy)-doped, and (f) (La, N)-doped SrTiO_3_. Figure S19: Figures of merit (ZT) versus temperature variation for (a) undoped, (b) La-doped, (c) Dy-doped, (d) N-doped, (e) (La, Dy)-doped, and (f) (La, N)-doped SrTiO_3_. Table S1: Amount of doping of La, Dy and N in SrTiO_3_. Table S2: Total energy (E_tot_), total energy per atom (E_atom_), and formation energy (E_form_) of the host sites replaced by La, Dy and N atoms. The number in the parentheses gives the total number of atoms in the bulk structure. The bold numbers for E_form_ representing the lowest values, indicating the most stable structures in experiments. Table S3: Lattice parameters of the most stable structures for undoped and doped SrTiO_3_ [43,44].
