# Supplementary material for: Improved Thermoelectric Properties of SrTiO3 via (La, Dy and N) Co-Doping: DFT Approach
Source: Molecules. 2022 Nov 16;27(22):7923. doi: 10.3390/molecules27227923 (PMC9693972; doi:10.3390/molecules27227923)
Supplement: Supplementary file 1 [file molecules-27-07923-s001.zip › molecules-2028105-supplementary.pdf]

Supporting Information

Improved Thermoelectric Properties of $SrTiO_3$ via (La, Dy and N) Co-Doping: DFT Approach

Pornsawan Sikam [1,2], Ruhan Thirayatorn [3], Thanayut Kaewmaraya [3,4], Prasit Thongbai [3,4], Pairot Moontragoon [3,4,5,*] and Zoran Ikonic [6]

1 Research Center for Quantum Technology, Faculty of Science, Chiang Mai University, Chiang Mai 50200, Thailand
2 Office of Research Administration, Chiang Mai University, Chiang Mai 50200, Thailand
3 Department of Physics, Khon Kaen University, Khon Kaen 40002, Thailand
4 Institute of Nanomaterials Research and Innovation for Energy (IN-RIE), Research Network of NANOTEC-KKU (RNN), Khon Kaen University, Khon Kaen 40002, Thailand
5 Thailand Center of Excellence in Physics, Commission on Higher Education, Bangkok 10400, Thailand
6 School of Electronic and Electrical Engineering, University of Leeds, Leeds LS2 9JT, UK
* Correspondence: mpairo@kku.ac.th

Table S1. Amount of doping of La, Dy and N in $SrTiO_3$.

Systems	Number of atoms						Chemical formula
	Sr	Ti	O	La	Dy	N	
Pristine $SrTiO_3$	8	8	24	-	-	-	$SrTiO_3$
12.5% of La-doped $SrTiO_3$	7	8	24	1	-	-	$Sr_{0.875}La_{0.125}TiO_3$
12.5% of Dy-doped $SrTiO_3$	7	8	24	-	1		$Sr_{0.875}Dy_{0.125}TiO_3$
4.2% of N-doped $SrTiO_3$	8	8	23	-	-	1	$SrTiO_{2.958}N_{0.042}$
(La, Dy)-co doped $SrTiO_3$	6	8	24	1	1	-	$Sr_{0.750}La_{0.125}Dy_{0.125}TiO_3$
(La, N)-co doped $SrTiO_3$	7	8	23	1	-	1	$Sr_{0.875}La_{0.125}TiO_{2.958}N_{0.042}$

Table S2. Total energy (E_{tot}), total energy per atom (E_{atom}), and formation energy (E_{form}) of the host sites replaced by La, Dy and N atoms. The number in the parentheses gives the total number of atoms in the bulk structure. The bold numbers for E_{form} representing the lowest values, indicating the most stable structures in experiments.

Bulk structures	E_{tot} (eV)	E_{atom} (eV)
Sr (2 Atoms)	-3.08	-1.54
Ti (3Atoms)	-23.59	-7.86
O (8 Atoms)	-39.52	-4.94
La (4 Atoms)	-20.65	-5.16

Dy (3 atoms)	-13.63	-4.54
N (2 atoms)	-16.54	-8.27
Undoped STO	-320.80	

**Structure**	**E_{tot} (eV)**	**E_{form} (eV)**
**La-doped STO**		
1. Sr replaced by La	-325.17	**-0.75**
2. Ti replaced by La	-312.41	5.69
3. O replaced by La	-307.65	13.38
**Dy-doped STO**		
1. Sr replaced by Dy	-323.87	**-0.06**
2. Ti replaced by Dy	-314.44	3.04
3. O replaced by Dy	-307.76	12.64
**N-doped STO**		
1. Sr replaced by N	-312.02	15.52
2. Ti replaced by N	-305.05	16.16
3. O replaced by N	-318.84	**5.29**

The formation energy is calculated using the equation

$E_f = E_{doped\ STO} - E_{STO} - nE_{doped\ atom} + nE_{host}$,

where $E_{doped\ STO}$, E_{STO}, $E_{doped\ atom}$, and E_{host} donate the total energy of the doped STO, the undoped STO, the atom doping into STO calculated from bulk structures, and the host atom doping calculated from bulk structures, respectively. Moreover, *n* is the number of atoms added or removed from the pristine STO to design the (La, Dy and N)-mono-doped STO.[1, 2].

Table S3. Lattice parameters of the most stable structures for undoped and doped $SrTiO_3$.

**Systems**	**a (Å)**	**b (Å)**	**c (Å)**	**alpha (°)**	**beta (°)**	**gamma (°)**
Pristine $SrTiO_3$	7.880	7.880	7.880	90.00	90.00	90.00
12.5% of La-doped $SrTiO_3$	7.883	7.883	7.883	90.00	90.00	90.00
12.5% of Dy-doped $SrTiO_3$	7.863	7.863	7.863	90.00	90.00	90.00
4.2% of N-doped $SrTiO_3$	7.889	7.889	7.900	90.00	90.00	90.00
(La, Dy)-co doped $SrTiO_3$	7.851	7.873	7.873	90.00	90.00	90.00
(La, N)-co doped $SrTiO_3$	7.876	7.906	7.906	89.76	90.00	90.00

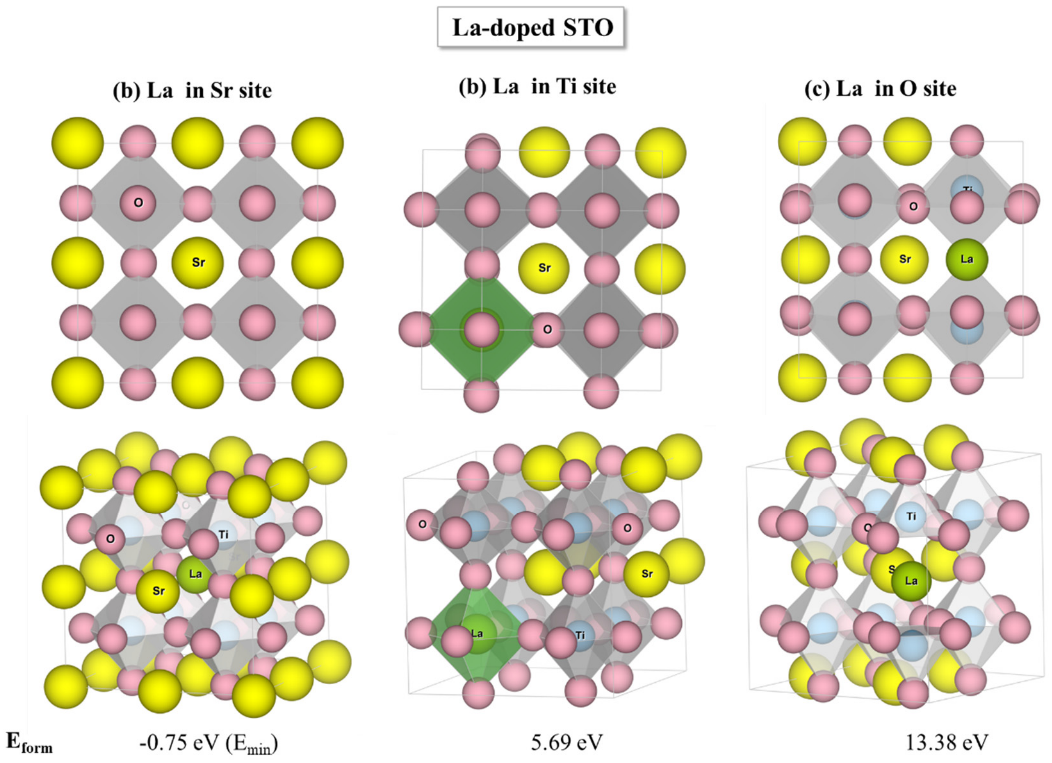

Figure S1. The optimized structures of La-doped $SrTiO_3$ after replacing La atoms in Sr, Ti, and O sites with formation energy (E_{form}) as the total energy as listed in Table S2. The pink spheres are the O atoms, yellow are Sr, light blue are Ti, and green are La. E_{min} indicates the structure providing the lowest formation energy.

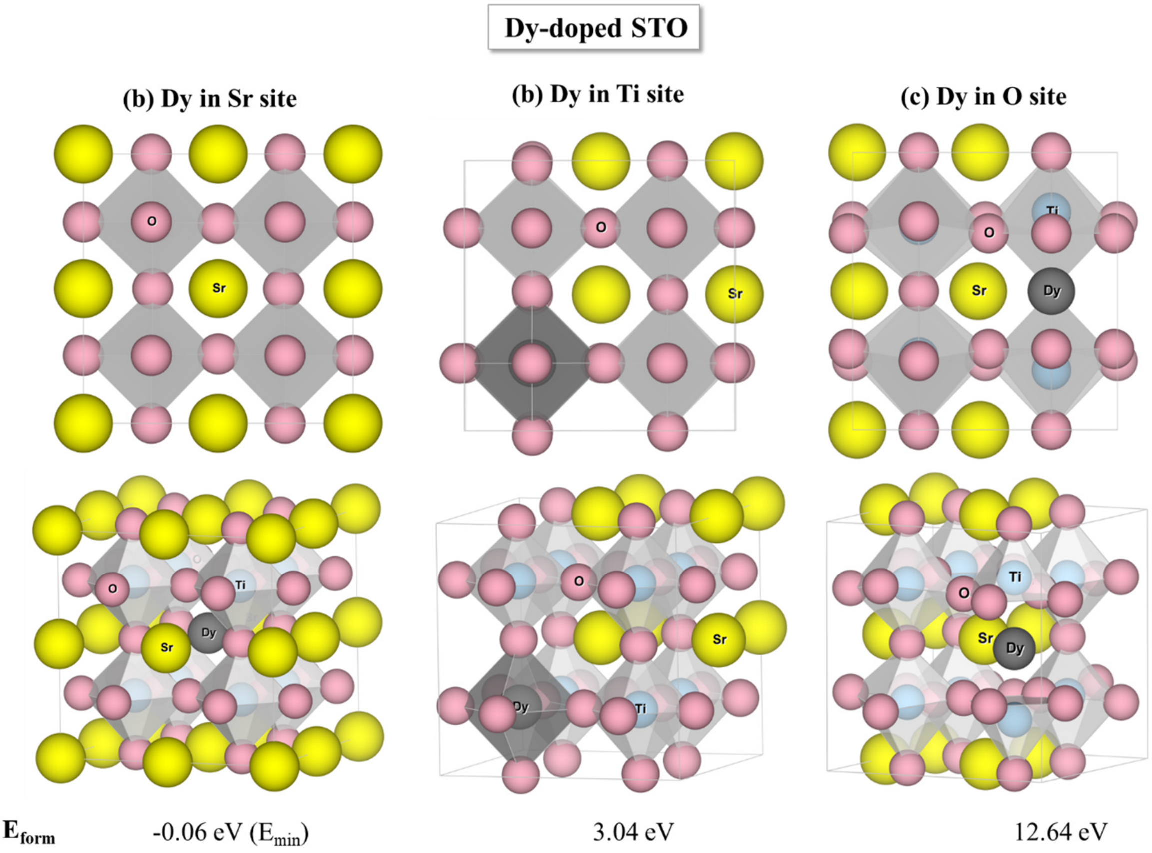

Figure S2. The optimized structures of Dy-doped $SrTiO_3$ after replacing Dy atoms in Sr, Ti, and O sites with formation energy (E_{form}) as the total energy as listed in Table S2. The pink spheres are the O atoms, yellow are Sr, light blue are Ti, and grey are Dy. E_{min} indicates the structure providing the lowest formation energy.

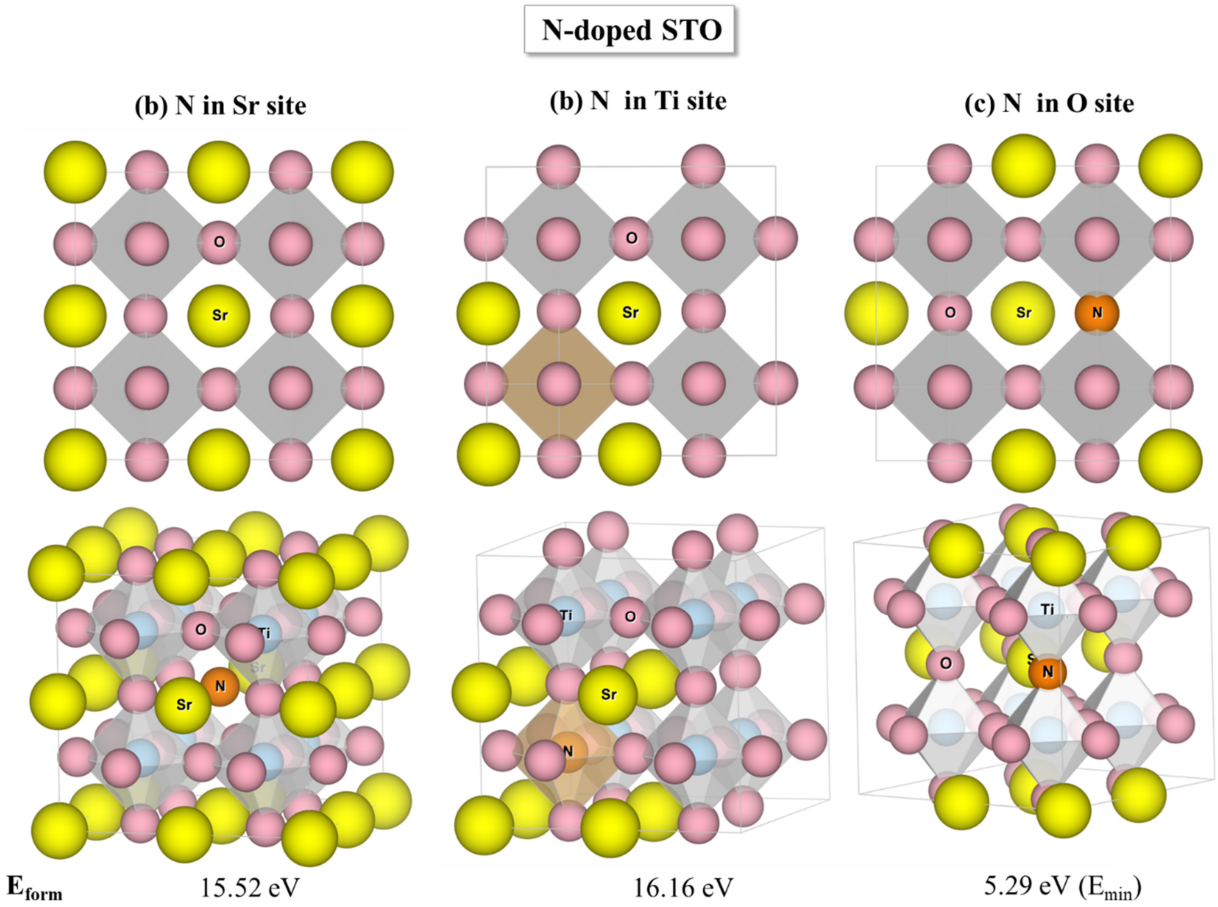

Figure S3. The optimized structures of N-doped $SrTiO_3$ after replacing N atoms in Sr, Ti, and O sites with formation energy (E_{form}) as the total energy as listed in Table S2. The pink spheres are the O atoms, yellow are Sr, light blue are Ti, and orange are N. E_{min} indicates the structure providing the lowest formation energy.

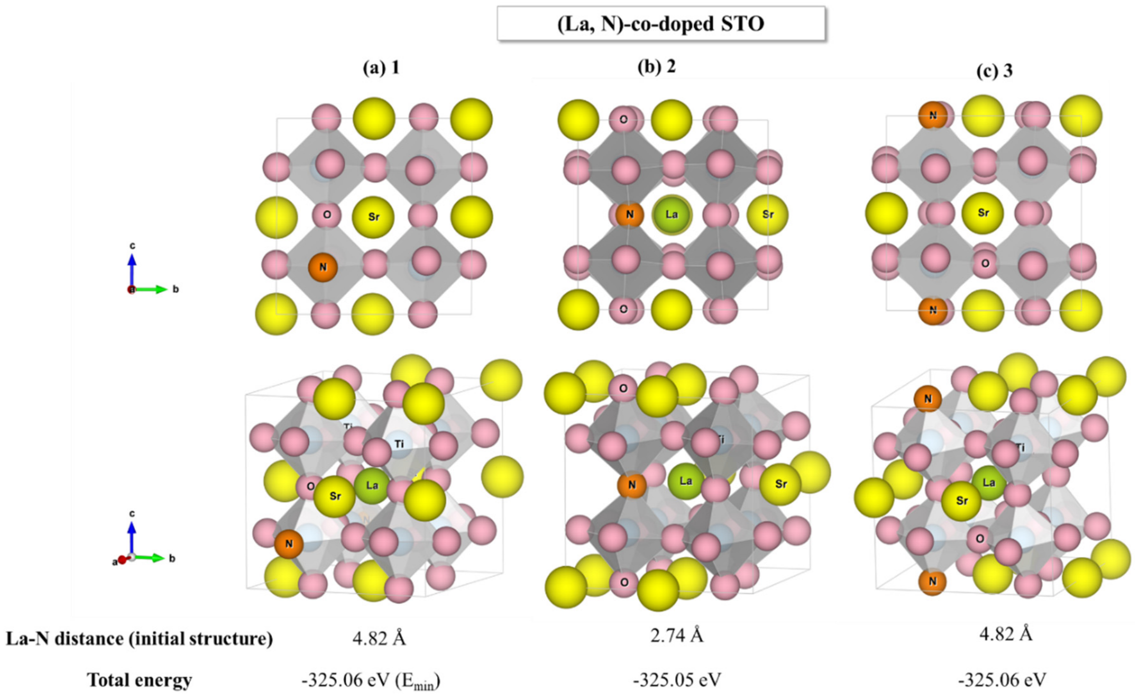

Figure S4. Three different La-N distances, with the total energy of the supercell configuration, for 3 different sites of La and N doping, in which both La and N atoms replace Sr and O sites, respectively. E_{min} indicates the structure providing the lowest total energy, which is the most stable structure.

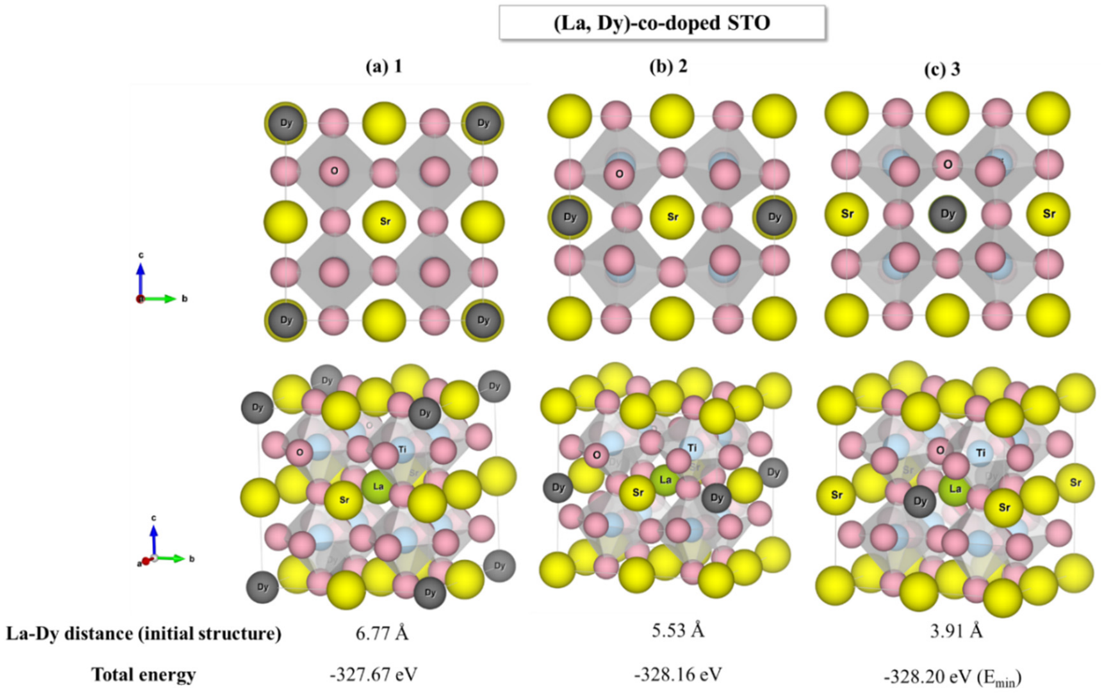

Figure S5. Three different La-Dy distances, with the total energy of the supercell configuration, for 3 different sites of La and Dy doping, in which both La and Dy atoms replace Sr sites. E_{min} indicates the structure providing the lowest total energy, which is the most stable structure.

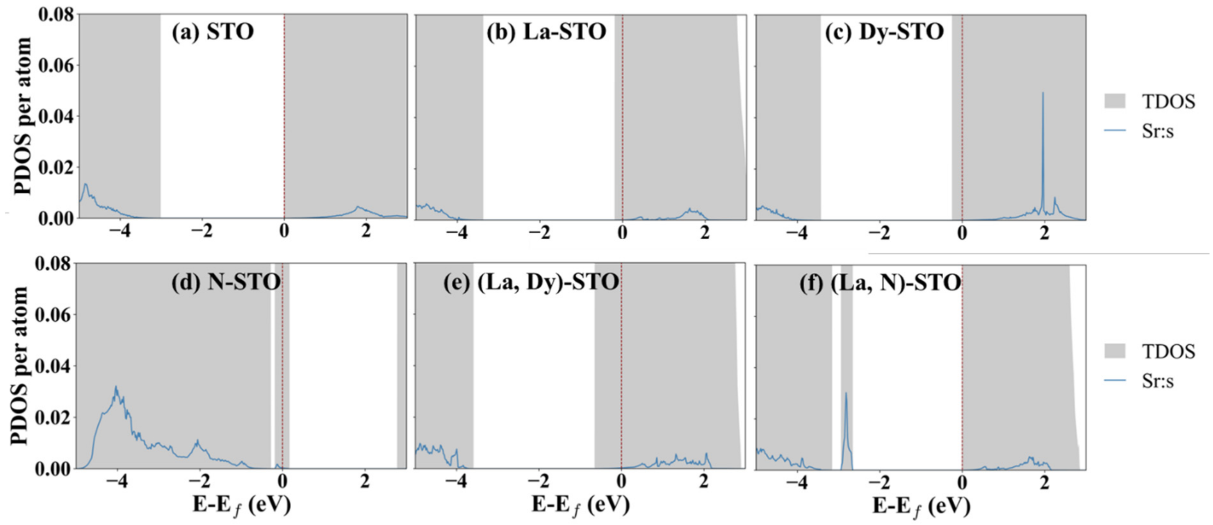

Figure S6. DOS of *s* orbitals of Sr for (a) undoped, (b) La-doped, (c) Dy-doped, (d) N-doped, (e) (La, Dy)-doped, and (f) (La, N)-doped $SrTiO_3$.

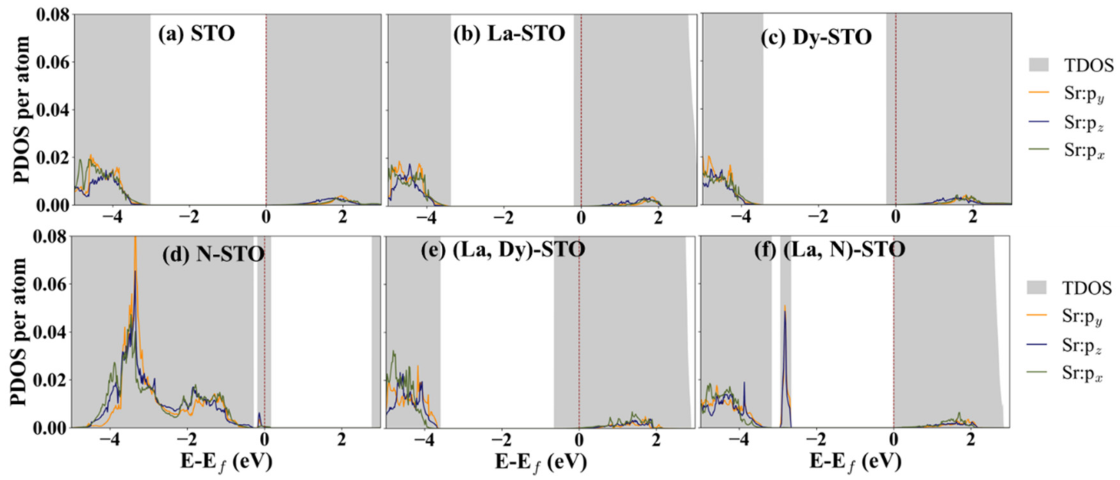

Figure S7. DOS of *p* orbitals of Sr for (a) undoped, (b) La-doped, (c) Dy-doped, (d) N-doped, (e) (La, Dy)-doped, and (f) (La, N)-doped $SrTiO_3$.

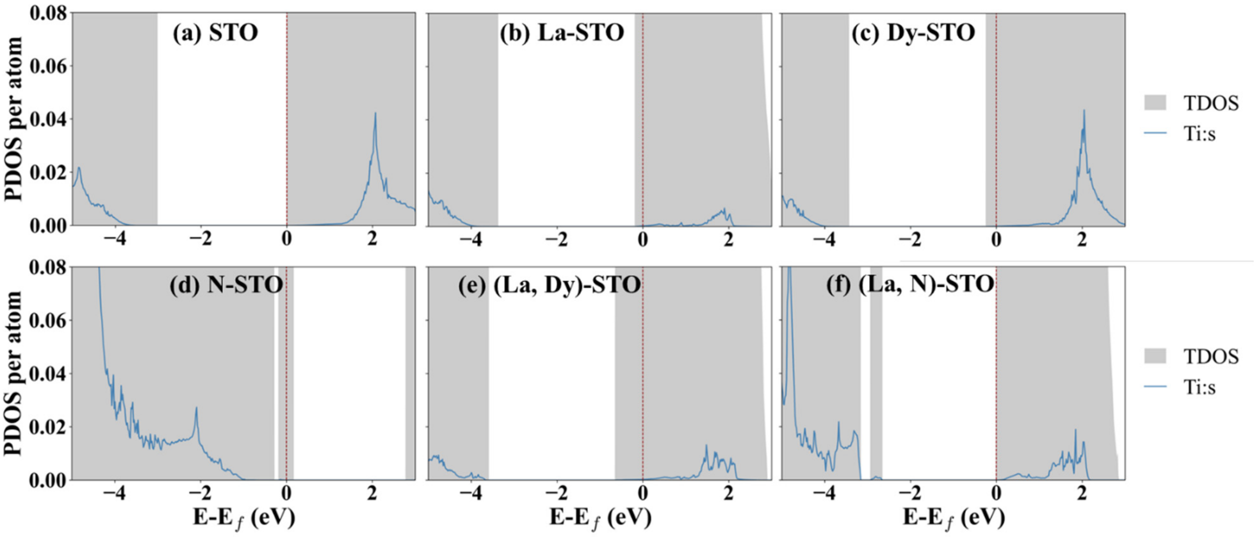

Figure S8. DOS of *s* orbitals of Ti for (a) undoped, (b) La-doped, (c) Dy-doped, (d) N-doped, (e) (La, Dy)-doped, and (f) (La, N)-doped $SrTiO_3$.

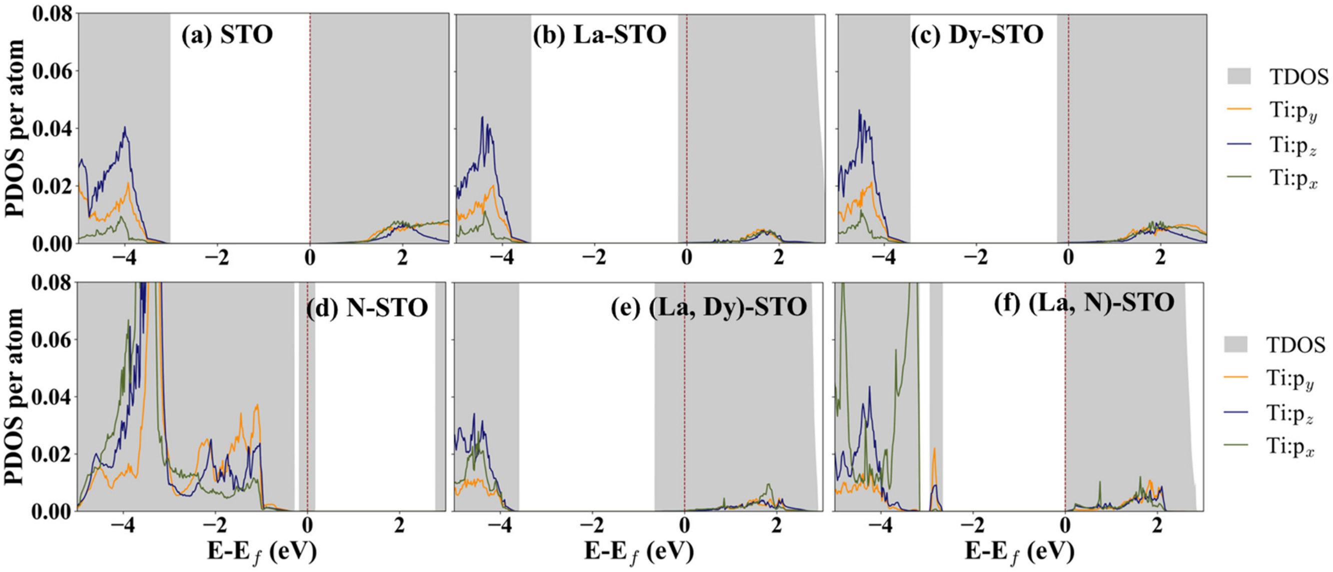

Figure S9. DOS of *p* orbitals of Ti for (a) undoped, (b) La-doped, (c) Dy-doped, (d) N-doped, (e) (La, Dy)-doped, and (f) (La, N)-doped $SrTiO_3$.

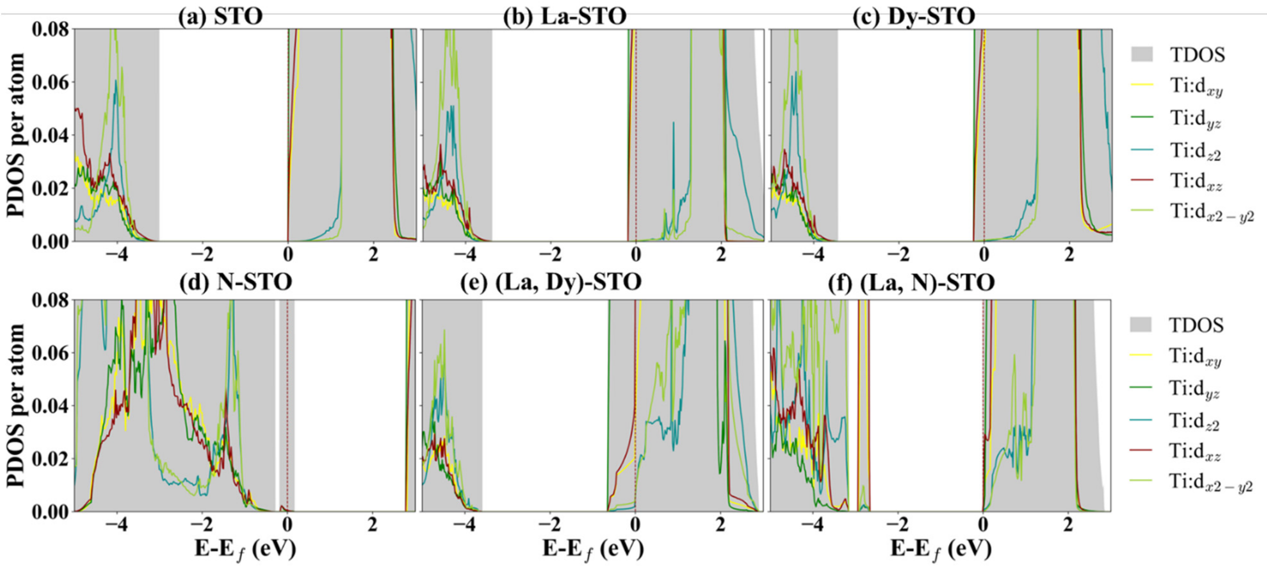

Figure S10. DOS of *d* orbitals of Ti for (a) undoped, (b) La-doped, (c) Dy-doped, (d) N-doped, (e) (La, Dy)-doped, and (f) (La, N)-doped $SrTiO_3$.

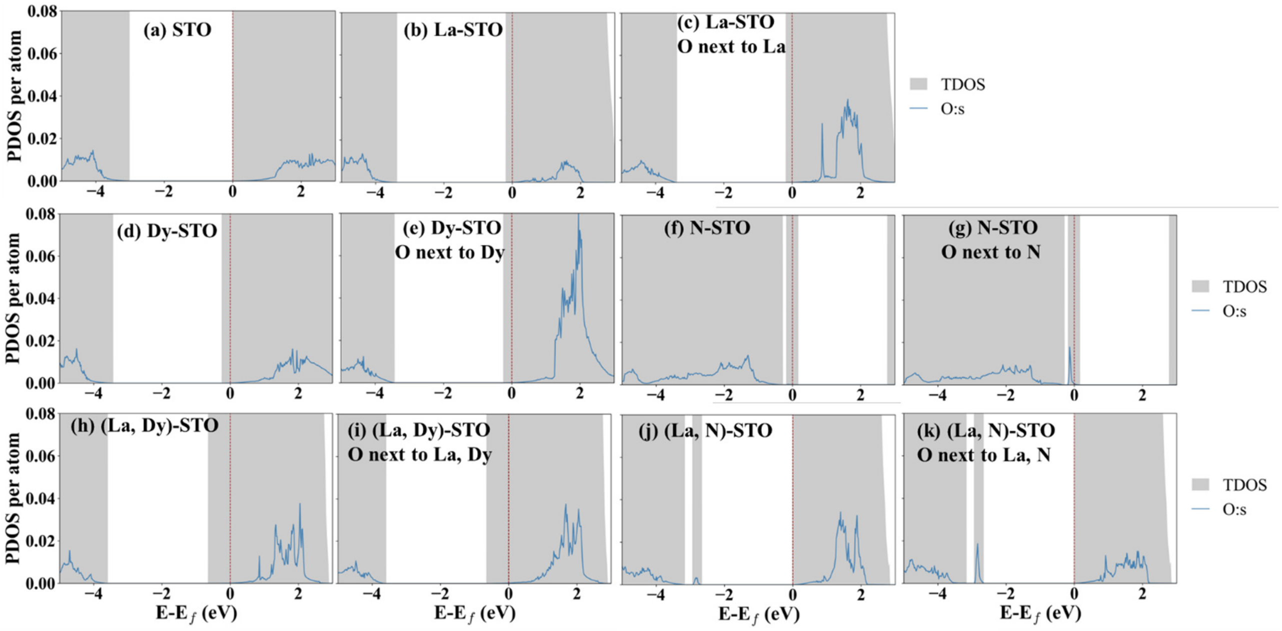

Figure S11. DOS of *s* orbitals of O for (a) undoped, (b, c) La-doped, (d, e) Dy-doped, (f, g) N-doped, (h, i) (La, Dy)-doped, and (j, k) (La, N)-doped $SrTiO_3$. Two O atoms are observed at positions which are (a, b, d, f, h, and j) remote from and (c, e, g, i, and k) neighboring the doped atoms.

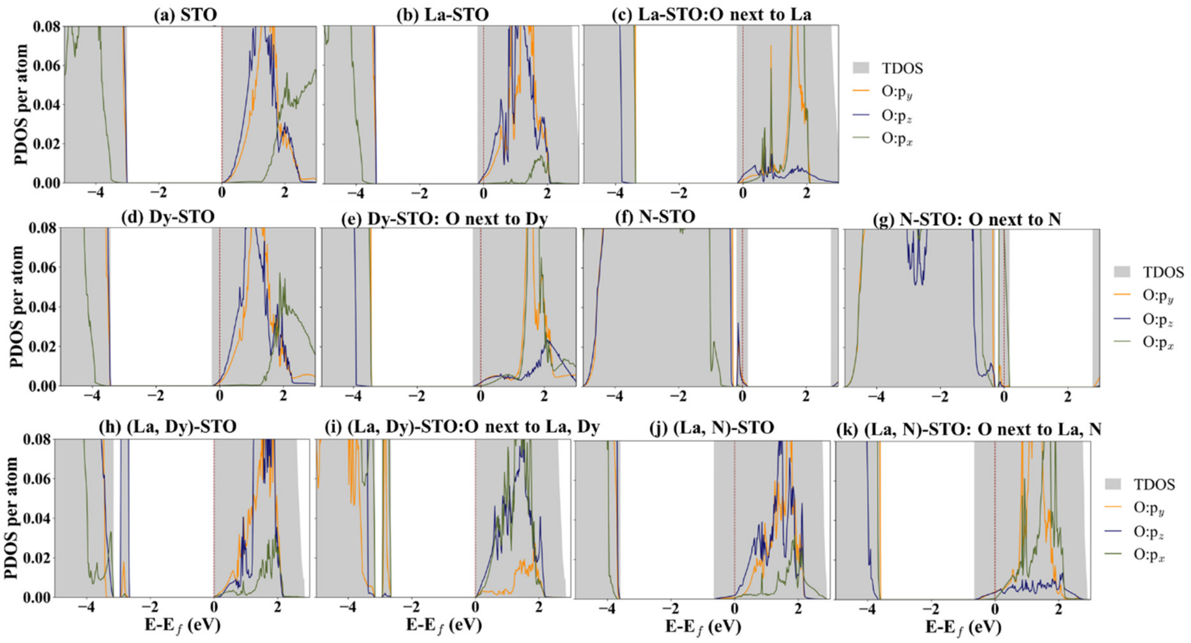

Figure S12. DOS of *p* orbitals of O for (a) undoped, (b, c) La-doped, (d, e) Dy-doped, (f, g) N-doped, (h, i) (La, Dy)-doped, and (j, k) (La, N)-doped $SrTiO_3$. Two O atoms are observed at positions which are (a, b, d, f, h, and j) remote from and (c, e, g, i, and k) neighboring the doped atoms.

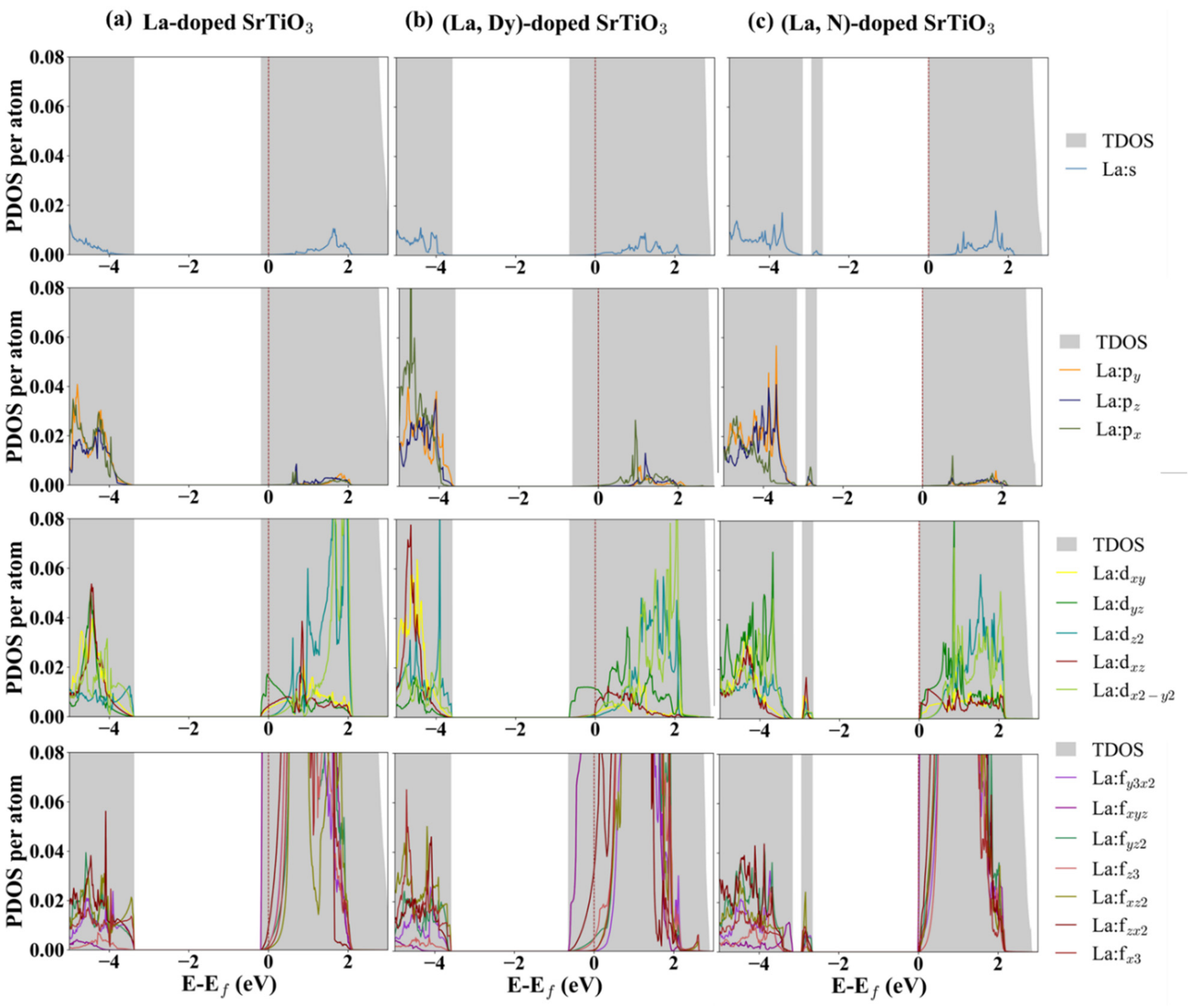

Figure S13. DOS of *s*, *p*, *d* and *f* orbitals of La for (a) La-doped, (b) (La, Dy)-doped, and (c) (La, N)-doped $SrTiO_3$.

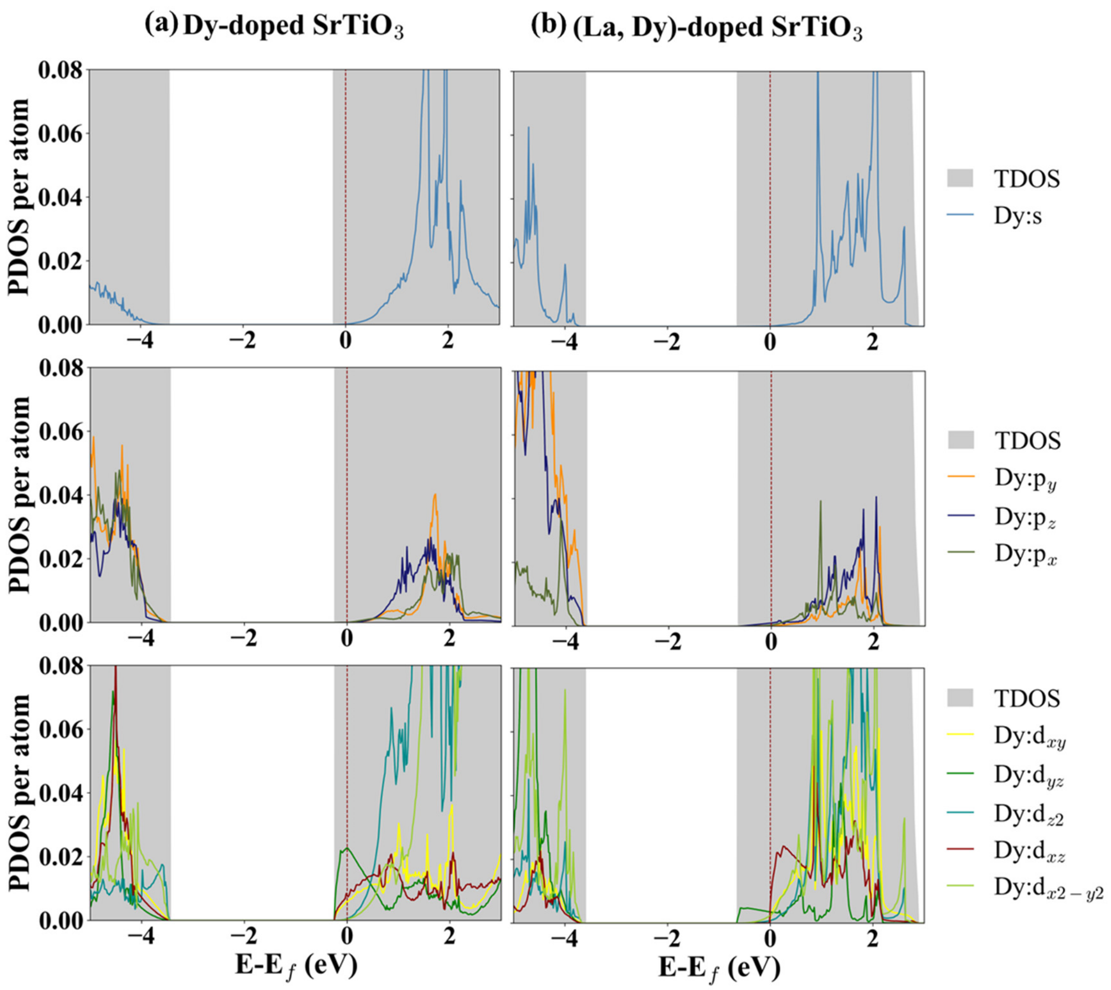

Figure S14. DOS of *s*, *p*, and *d* orbitals of Dy for (a) Dy-doped and (b) (La, Dy)-doped $SrTiO_3$.

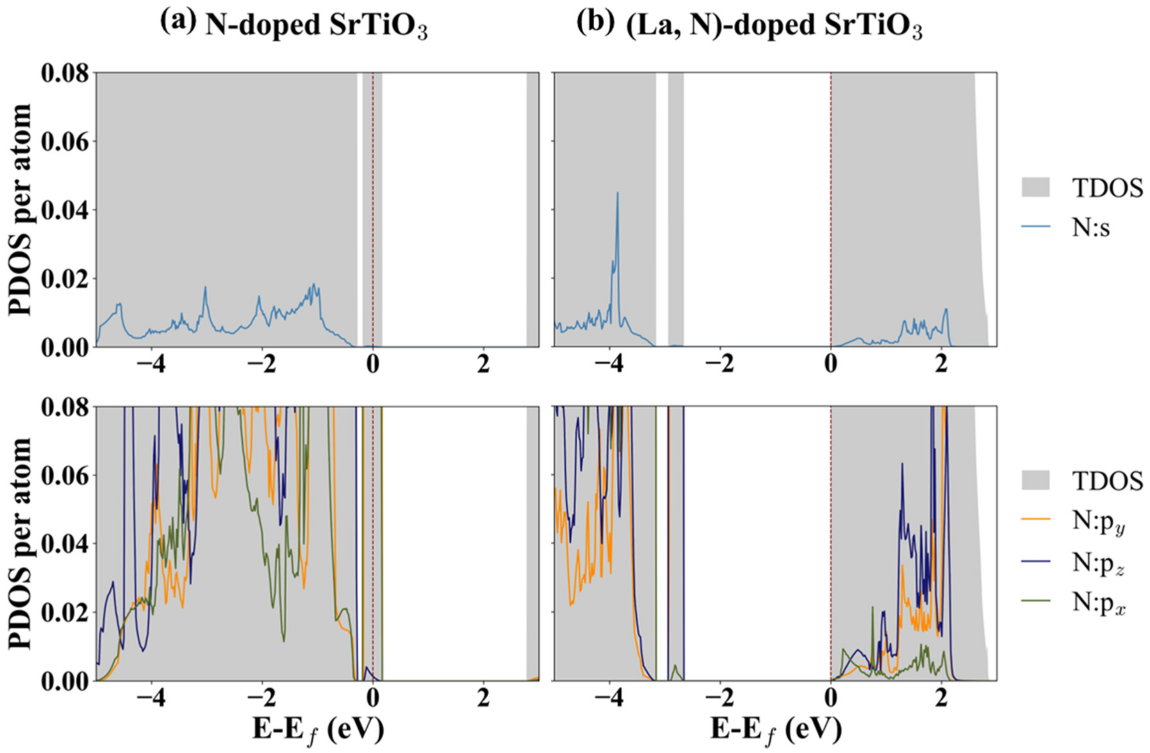

Figure S15. DOS of *s* and *p* orbitals of N for (a) N-doped and (c) (La, N)-doped $SrTiO_3$.

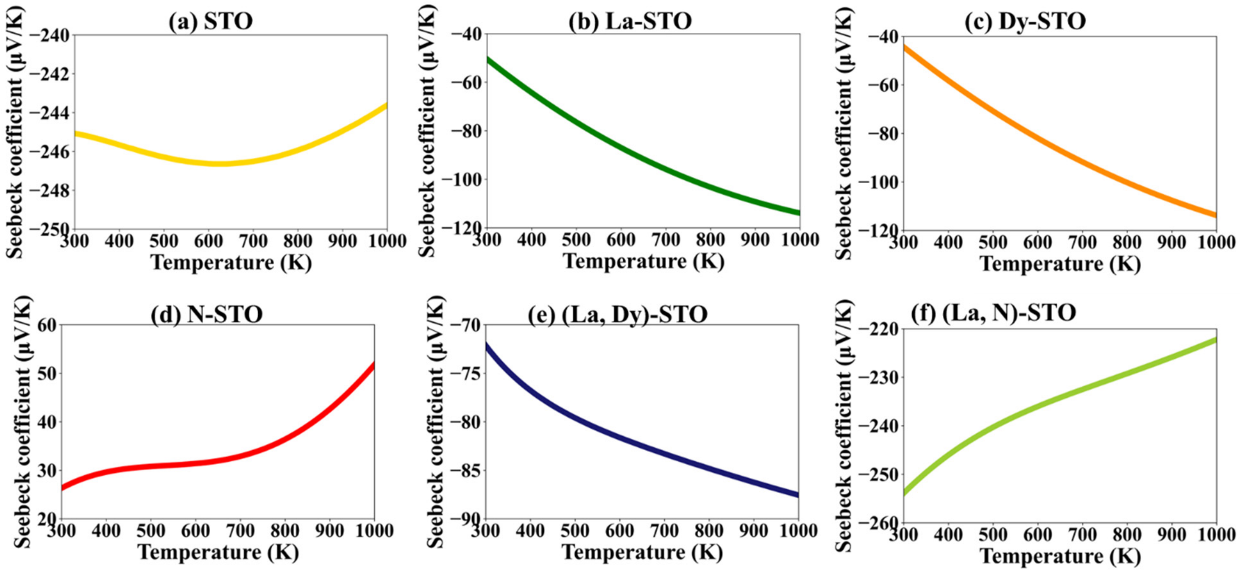

Figure S16. Seebeck coefficient versus temperature variation for (a) undoped, (b) La-doped, (c) Dy-doped, (d) N-doped, (e) (La, Dy)-doped, and (f) (La, N)-doped $SrTiO_3$.

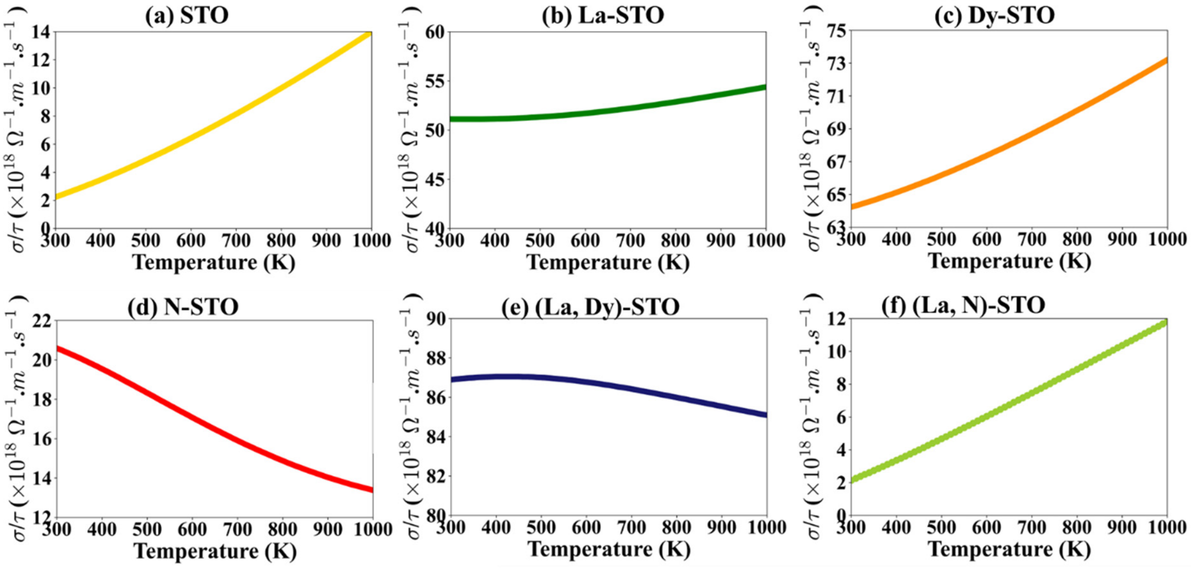

Figure S17. Ratio of electrical conductivity to relaxation time (σ/τ) with temperature variation for (a) undoped, (b) La-doped, (c) Dy-doped, (d) N-doped, (e) (La, Dy)-doped, and (f) (La, N)-doped $SrTiO_3$.

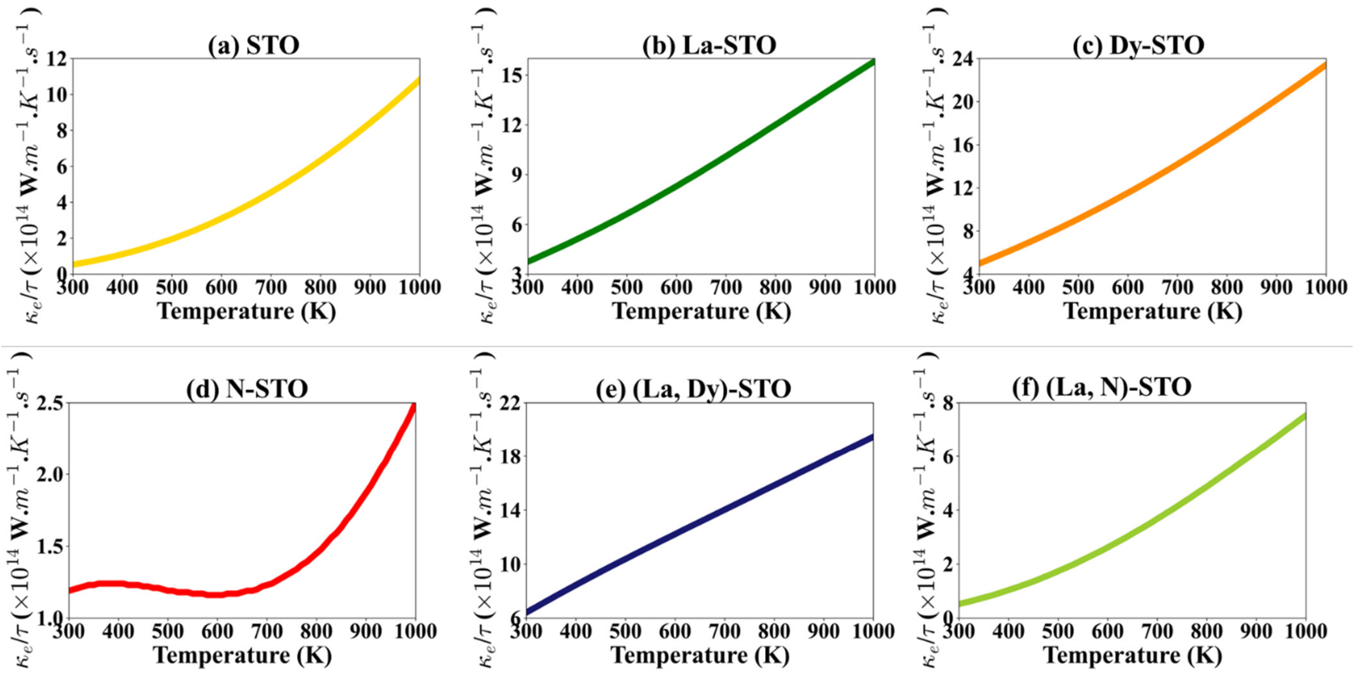

Figure S18. Ratio of electronic thermal conductivity to relaxation time (κ_e/τ) with temperature variation for (a) undoped, (b) La-doped, (c) Dy-doped, (d) N-doped, (e) (La, Dy)-doped, and (f) (La, N)-doped $SrTiO_3$.

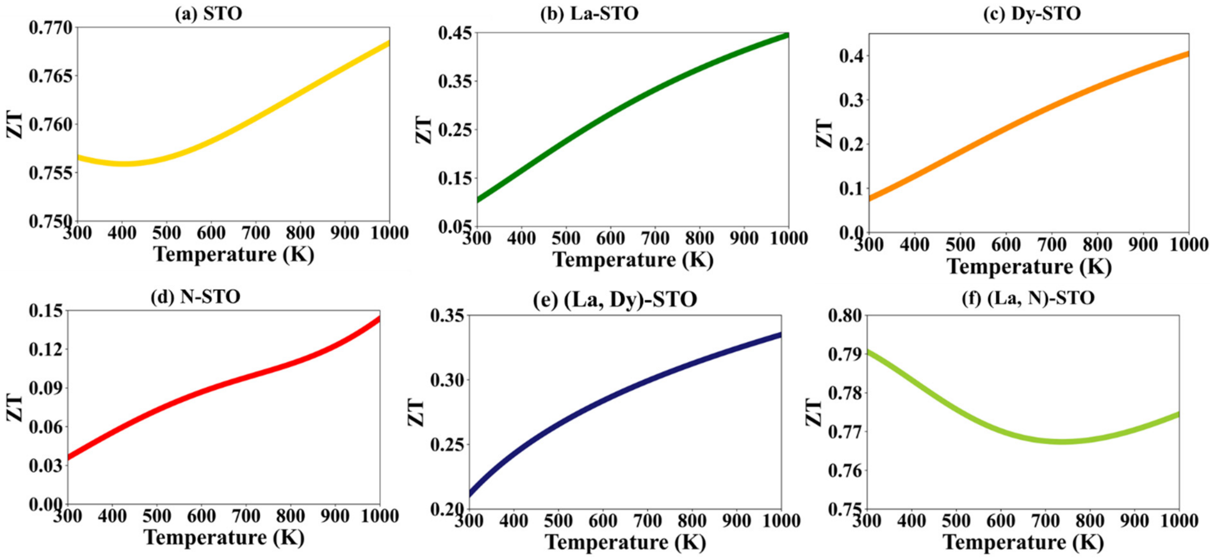

Figure S19. Figures of merit (ZT) versus temperature variation for (a) undoped, (b) La-doped, (c) Dy-doped, (d) N-doped, (e) (La, Dy)-doped, and (f) (La, N)-doped $SrTiO_3$.
